# Structural Origin of Semiconductor Optical Size Effect through In_2_O_3_ Nanocubes and Use in Photocatalytic Benzothiazole Formation

**DOI:** 10.1021/acs.inorgchem.6c01581

**Published:** 2026-05-28

**Authors:** Xin-Ru Lin, Kuo-Chang Chien, Bo-Hao Chen, Michael H. Huang

**Affiliations:** † Department of Chemistry, 34881 National Tsing Hua University, Hsinchu 300044, Taiwan; ‡ National Synchrotron Radiation Research Center, Hsinchu 300092, Taiwan

## Abstract

In_2_O_3_ nanocubes with sizes of 10, 72, and 95 nm have been synthesized solvothermally in ethanol. Synchrotron X-ray diffraction (XRD) patterns reveal ultrasmall peak shifts among these samples. Moreover, while 10 nm cubes give symmetric diffraction peaks, those of 72 and 95 nm cubes can be deconvoluted into bulk and surface layer lattice components. Fast Fourier transform (FFT) processing of their high-resolution transmission electron microscopy (HR-TEM) images presents similar lattice point appearances throughout the crystal for a 10 nm cube, while 72 and 95 nm nanocubes show greater surface lattice deviations compared with inner lattice points. These lattice differences should be the structural basis for the observed optical size effect, with band gaps of 10 and 95 nm cubes differing by 0.13 eV. Heating the nanocubes to high temperatures changed their color from white to yellowish due to cell constant changes and significant lattice point distortion. This simple experiment demonstrates that lattice variations can produce color changes in semiconductor crystals. The In_2_O_3_ nanocubes were employed to photocatalyze the condensation of 2-aminothiophenol and benzaldehyde, forming benzothiazole. Active species have been identified. Both intrinsic and thermally induced lattice deviations can cause light absorption shifts, explaining why the same semiconductor material displays diverse colors.

## Graphical abstract

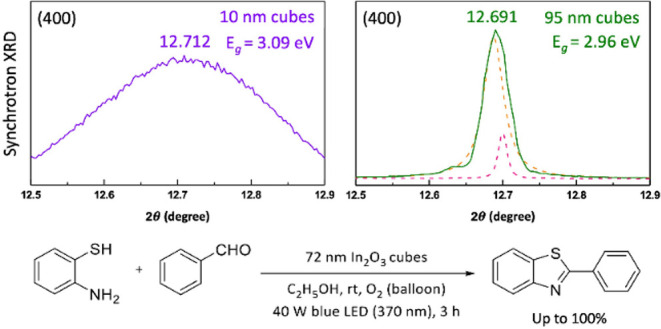

## Introduction

Semiconductor crystals broadly exhibit facet-related electrical conductivity, photocatalytic activity, and light absorption behaviors.^1–5^ Moreover, piezoelectricity, magnetism, and thermoelectric responses also display facet dependence.^6–10^ Through synchrotron XRD pattern analysis and FFT treatment of HR-TEM images, the emergence of all these phenomena can be traced to the presence of a facet-related layer below the crystal surface with considerable lattice point distortions, in addition to slight bulk cell constant changes for different particle shapes.^3,8–11,16^ These structural variations affect charge transport and light absorption, as well as various other material properties. This lattice reality in ionic solids arise naturally as required by thermodynamics and from the temperature-induced lattice disturbance. In terms of facet-dependent optical behavior, Cu_2_O crystals with similar particle volumes still show distinctly different colors.^12^ While optical facet effect can now be understood, semiconductor crystals also show intriguing size-related and continuous absorption band red-shifts with increasing particle dimensions from quantum dots to microcrystals, including Cu_2_O, Ag_2_O, Fe_3_O_4_, and BaZrO_3_ polyhedra.^3,9,12–17^ Optical size effect is likely also related to slight cell constant changes. Previously, MnS nanocubes and octahedra have revealed slight size-related XRD peak shifts.^18^ To better address the optical size effect, it is highly desirable to synthesize nanocrystals ranging from below 10 nm to beyond 100 nm. Polyhedral In_2_O_3_ particles with a broad size range have been reported.^19–21^ In_2_O_3_ quantum dots with sizes below 2 nm have also been prepared.^22^ In_2_O_3_ is an n-type semiconductor. Cubic In_2_O_3_ has a bixbyite crystal structure.^23^ By heating indium acetylacetonate (In­(acac)_3_) with oleylamine at 240 °C for 12 h, 15 nm In_2_O_3_ cubes were obtained.^19^ Hollow In_2_O_3_ structures with band gaps of 3.05–3.30 eV displayed photocatalytic activity toward dye degradation.^24^ Size-tunable In_2_O_3_ cubes still have not been reported. Another intriguing optical phenomenon for many semiconductor materials is that they exhibit diverse colors. For In_2_O_3_ powder, its color can vary from white to yellowish. No study has attempted to address this observation. Again this phenomenon can be understood, recognizing that a certain degree of lattice distortion is inherent in these crystals. Since temperature is a major factor in the industrial preparation of oxide powder, annealing the prepared In_2_O_3_ particles to induce greater lattice distortion may change its color.

In addition to growing size-tunable In_2_O_3_ nanocrystals, we also consider utilizing the particles to photocatalyze organic transformation. A coupling reaction between 2-aminothiophenol and benzaldehyde to generate benzothiazole is attractive, since benzothiazole derivatives have exhibited pharmacological activities, including antitumor and antiseptic effects.^25^ Various approaches have been adopted to prepare benzothiazoles.^25,26^ Covalent organic frameworks have also been employed for photocatalytic generation of benzothiazole and benzimidazole compounds.^27,28^ However, photocatalyzed benzothiazole formation using oxide nanocrystals appears not yet reported.

In this study, size-tunable In_2_O_3_ nanocubes have been synthesized solvothermally. Synchrotron XRD analysis indicates better peak fitting with two lattice components for larger cubes. FFT-processed HR-TEM images also reveal greater surface lattice deviations for the larger cubes. The structural differences between small and larger nanocubes offer insights of the absorption band shifts. Interestingly, annealing the In_2_O_3_ nanocubes produced a visible change to their color from significant lattice disturbance. The In_2_O_3_ nanocubes were used to photocatalyze a condensation reaction between 2-aminothiophenol and benzaldehyde forming benzothiazole. Active species trapping experiments were conducted to elucidate the reaction mechanism.

## Results and Discussion

### Synthesis and Structural Characterization of In_2_O_3_ Nanocubes

For In_2_O_3_ nanocrystal growth, ethanol was chosen as the solvent and sodium hydroxide as the base. By tuning the amount of base, reaction temperature, and time, In_2_O_3_ nanocubes with different sizes were successfully synthesized. These parameters were optimized to produce uniform nanocubes. Different reaction vessels were used to achieve particle size and shape control. The reaction equation should be 2In^3+^ + 4OH^–^ → In_2_O_3_ + H_2_O + 2H^+^. With only ethanol and NaOH, the solution pH was 12.58. Upon adding indium nitrate, a white precipitate was observed and the pH decreased to 12.30. After heating the solution in an oven, the pH was 12.49, due to the greater amount of NaOH introduced for crystal growth. Figure 1 gives TEM and scanning electron microscopy (SEM) images of the synthesized In_2_O_3_ nanocubes with average edge lengths of 10, 72, and 95 nm. The particles appear to be separated without clustering. Their size distribution histograms are available in Figure S1. Normal Gaussian size distribution curves were obtained for these samples. The 10 nm sample contains many particles smaller than 10 nm, so their sizes approach the quantum dot domain.

**Figure 1:**
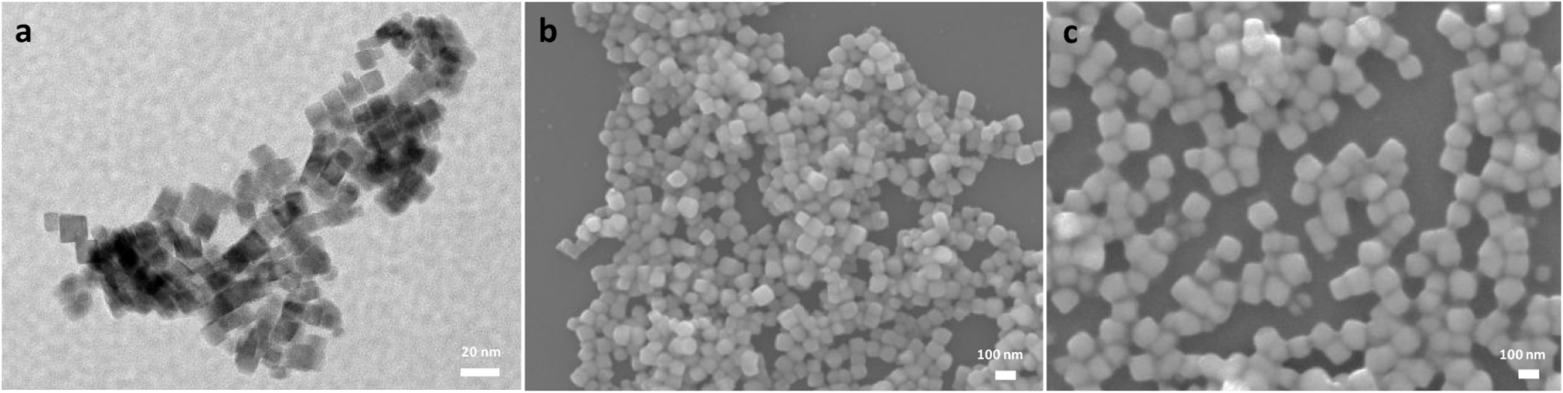
(a) TEM and (b, c) SEM images of the synthesized In_2_O_3_ nanocubes with average sizes of (a) 10, (b) 72 and (c) 95 nm.

Figure 2 gives in-house XRD patterns of these In_2_O_3_ samples, matching well with the reference and reported patterns.^29^ The 10 nm cube sample shows notably broader peak widths from the small particle sizes, and yields lower peak intensities. A gradual diffraction peak shift toward lower angles is observed as the particle size increases. This means the unit cell constant expands slightly with increasing nanocube size. For more precise analysis of the structural variation, synchrotron XRD measurements were conducted. Synchrotron radiation offers high photon flux, excellent monochromaticity, and tunable wavelength to significantly enhance the resolution and signal-to-noise ratio of diffraction peaks. These features are particularly important for subtle lattice variation detection. At a synchrotron X-ray wavelength of 0.56025 Å, the total X-ray absorption for In_2_O_3_ loaded in a 0.2 mm capillary tube was calculated to be 0.65. This absorption is sufficiently low, preventing a reduction in diffraction peak intensity.

**Figure 2:**
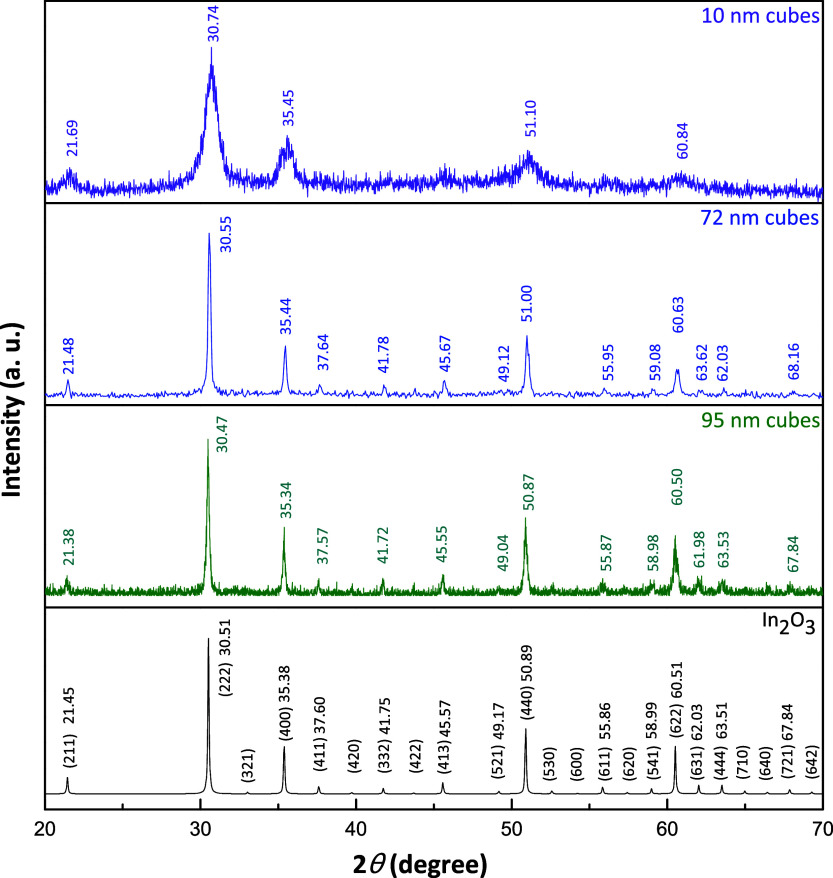
In-house XRD patterns of the size-tunable In_2_O_3_ nanocubes. The peak widths increase with decreasing particle sizes.

Figure S2 provides synchrotron XRD patterns of the In_2_O_3_ nanocubes. While the peak positions are mostly constant, a close inspection still reveals an ultrasmall shift in diffraction peaks toward lower angles as the particle size increases, indicating an expanded lattice constant for the larger nanocubes. Figure 3 presents the expanded synchrotron XRD peaks of In_2_O_3_ nanocubes to identify the tiny peak shifts. Moreover, while 10 nm In_2_O_3_ cubes have symmetric peaks, larger cubes display some peak asymmetry, particularly for the 95 nm sample. This observation suggests that In_2_O_3_ nanoparticles may develop into two distinct structural regions, corresponding to the bulk and surface domains, with increasing particle sizes. The measured synchrotron XRD patterns, along with the reported crystal structure of In_2_O_3_, were imported into the GSAS-II software for Rietveld refinement (Figure S3). To minimize potential errors caused by sample placement, a zero-shift correction was applied. The refinable parameters, including unit cell dimensions, crystal size, microstrain, thermal vibration, and unique axis, were adjusted to improve the agreement between the calculated and observed patterns. Using a single-phase model for diffraction peak fitting, the refinement yielded low *wR* and *R*(*F* ^2^) values, indicating a reliable and accurate fit of the structural model to the experimental data. However, the peak asymmetry observed in the diffraction patterns of 72 and 95 nm In_2_O_3_ cubes, along with the increasing values of *wR* and *R*(*F* ^2^) with particle size, a two-phase refinement model was found to achieve a better fit with the experimental data. As seen in Figure 3, the introduction of a second phase effectively compensates for the asymmetry observed in peaks. Table S1 lists the refined parameters. The unit cell parameter increases slightly with particle size, which is consistent with the peak shifts seen in the diffraction patterns. After refinement, the weight percentages of the bulk and surface components were obtained, allowing estimation of the surface shell thickness (Figure S4). The 10 nm cubes do not exhibit distinguishable bulk and surface regions. The 72 and 95 nm cubes have a thin surface layer with thicknesses of 1–1.7 nm. Thus, as In_2_O_3_ particle size increases from quantum dots to large nanocrystals, distinct bulk and surface lattice components begin to emerge.

**Figure 3:**
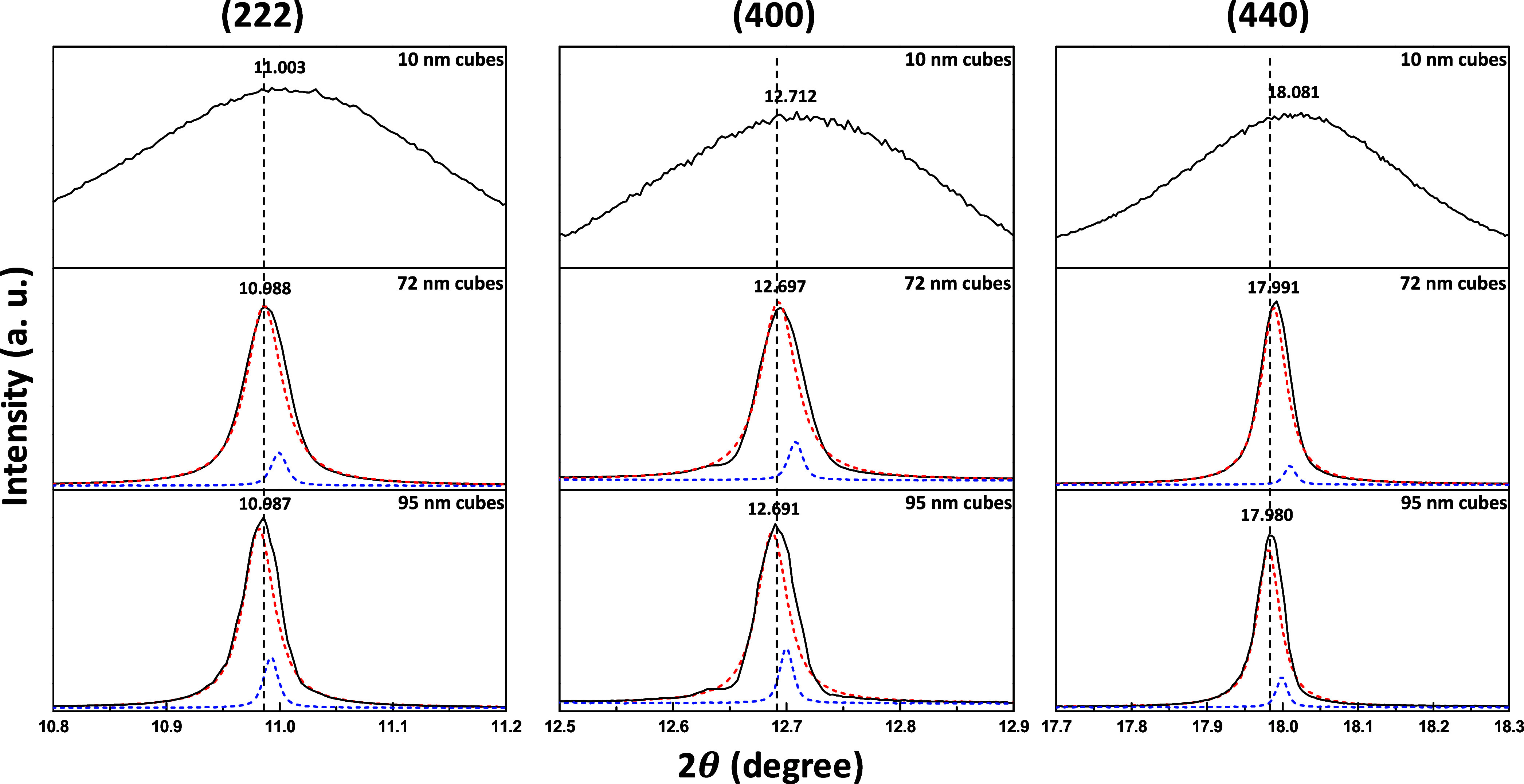
Expanded synchrotron XRD peaks of In_2_O_3_ nanocubes showing the bulk and surface layer lattice components from Rietveld refinement. The blue trace represents the surface layer component. The red trace indicates the bulk lattice component.

Figure S5 offers HR-TEM characterization of these nanocubes. The selected-area electron diffraction (SAED) patterns indicate single crystallinity of the nanocubes. All In_2_O_3_ cubes expose the same {100} surfaces. The (200) planes of the 10 nm cube give a *d*-spacing of 4.8 Å, while those of the 72 nm and 95 nm cubes show a larger *d*-spacing of 5.0 Å. The findings are consistent with an increased cell constant with bigger particles. Significantly, while normal (200) lattice fringes along the two perpendicular directions are observed for the 72 and 95 nm cubes, the 10 nm cube shows paired (200) planes along only one direction.

Next, lattice spot images were obtained by applying FFT processing to the HR-TEM images (Figure 4). Upon magnifying both the inner and outer regions of the lattice point images for the 10 nm cube, the spots in both regions appear elongated along the same direction, indicating a more uniform lattice environment throughout the particle. Lattice spot elongation results from considerable atomic position deviations accumulated over the entire length of the particle. The high reaction temperatures contribute to the lattice deviations. For the 72 nm and 95 nm cubes, the outer regions show more abundant lattice spots, when compared with the inner regions. The spots close to the surface appear significantly stretched along certain directions, while the interior lattice points are more rounded, revealing a clear distinction between the bulk and surface regions in larger particles. Hence, ultrasmall nanocrystals below 10 nm can exhibit strikingly different lattice features from those in larger crystals. This suggests that quantum dot emission is enhanced not simply because of the size effect, but lattice variations can actually occur.^30^

**Figure 4:**
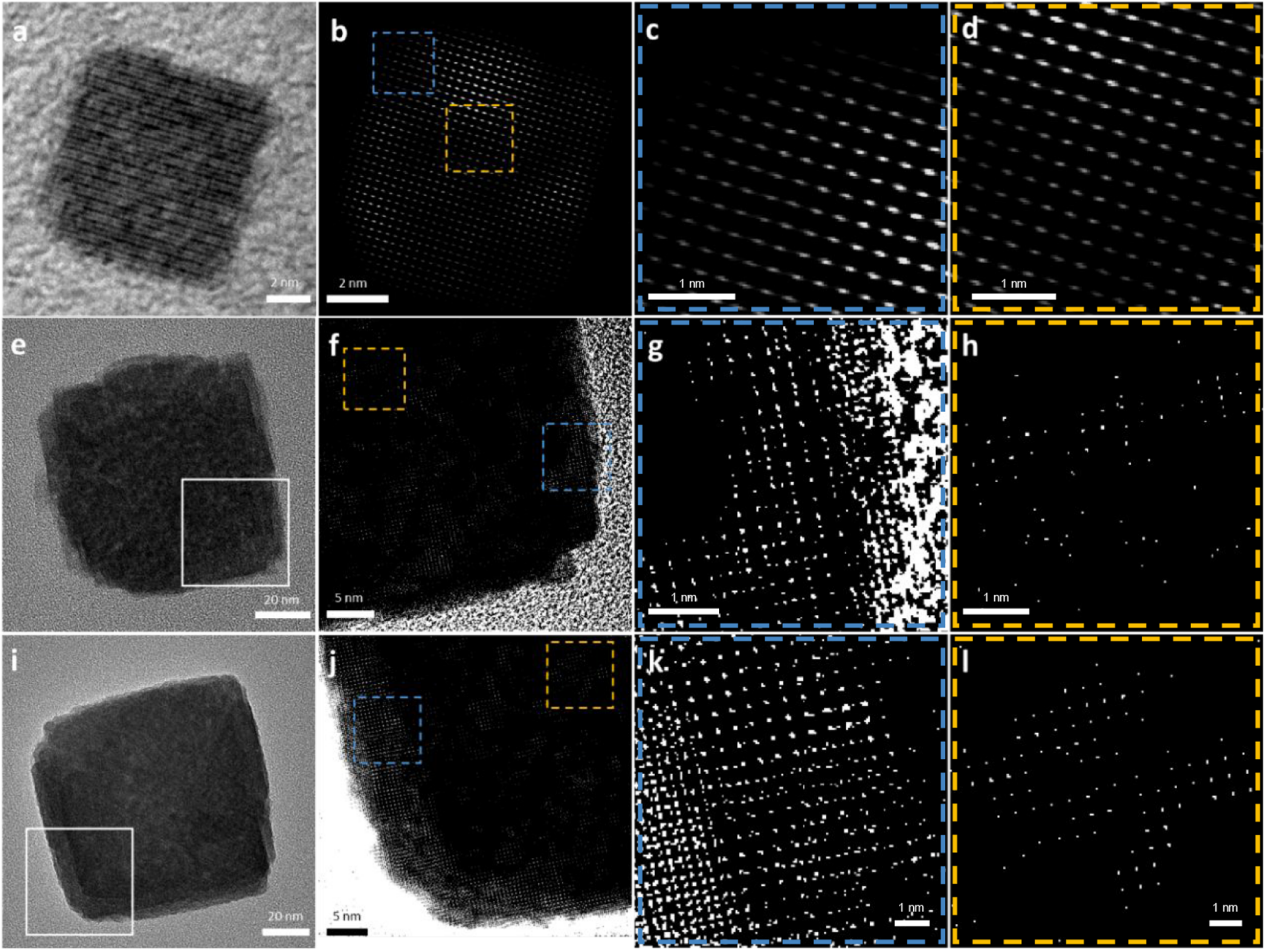
(a, e, i) HR -TEM images and (b, f, j) FFT-processed lattice point images of the white square regions for (a–d) 10, (e–h) 72, and (i–l) 95 nm In_2_O_3_ cubes. (c, g, k) Expanded blue square areas in the lattice point images. (d, h, l) Expanded yellow square areas in the lattice point images.

Figure 5 presents diffuse reflectance spectra and the corresponding Tauc plot of the In_2_O_3_ nanocubes. As expected, with decreasing particle size, absorption band is blue-shifted. The band gaps are 3.09, 2.99, and 2.96 eV for 10, 72, and 95 nm cubes, respectively. There is a considerable 0.13 eV difference between 10 and 95 nm In_2_O_3_ cubes. Thus, optical size effect should also be related to lattice variations and cell constant changes. The lattice deviations should accumulate with increasing particle sizes, so 72 and 95 nm cubes can still give somewhat different band gaps, despite having similar cell constants. Our understanding of quantum confinement effect is not ideal, since the effect of lattice deviations to light absorption is not considered. It is also not always suitable to assign a fixed value to a material as its bulk band gap; band gap can vary depending on the sample measured. A simple example to demonstrate this is by checking the diverse colors of Fe_2_O_3_ powder.

**Figure 5:**
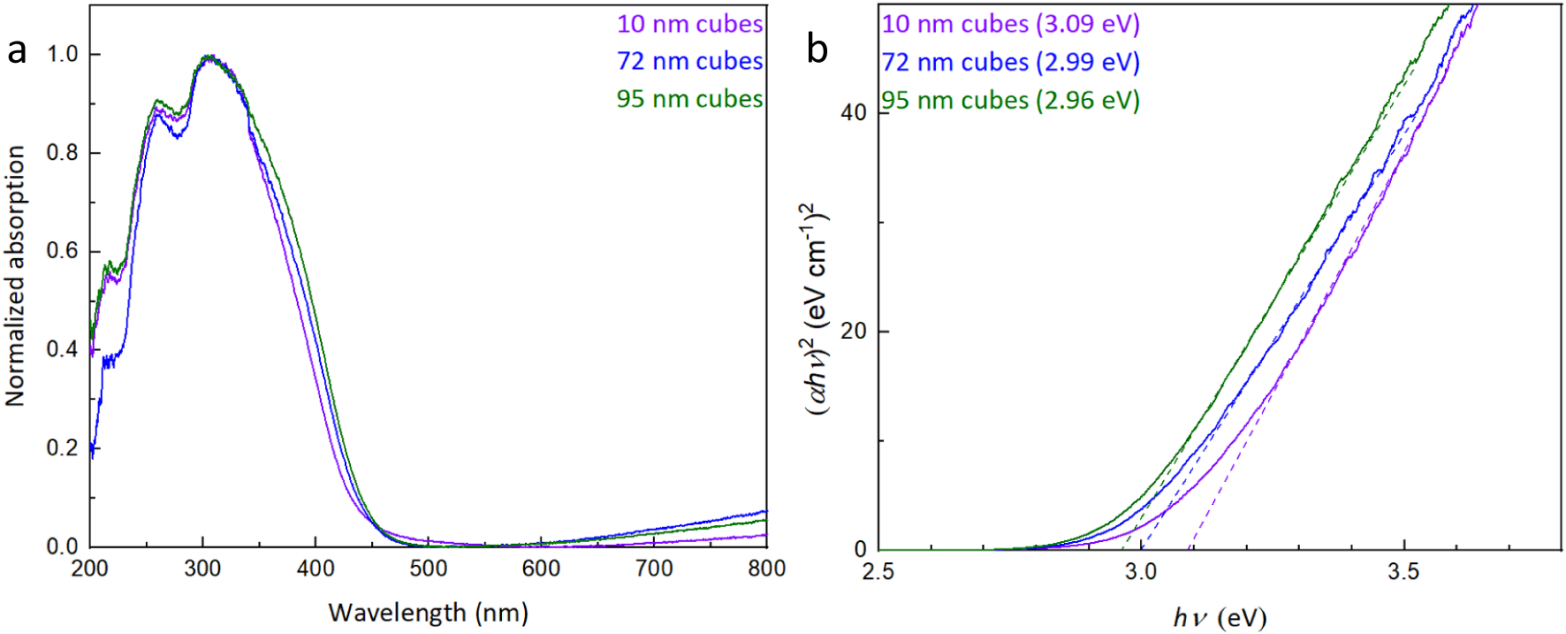
(a) Diffuse reflectance spectra and (b) the Tauc plot of In_2_O_3_ nanocubes.

Since the synthesized In_2_O_3_ nanocubes are nearly white in color, it was interesting to see if thermal treatment to cause significant lattice distortion can turn the particles yellowish. This would demonstrate that diverse colors seen in commercial In_2_O_3_ powder are linked to lattice variation. A larger amount of the 72 nm In_2_O_3_ cube sample was heat-treated to 500 °C for 2 h in an oven. Figure 6 shows the sample color has indeed changed to yellowish after annealing, supported by a notable absorption edge shift to longer wavelengths. SEM images indicate that the cubic particle shape is still recognizable after heating (Figure S6). XRD patterns reveal peak shifts to higher angles from slight lattice contraction (0.23%) after annealing. Figure S7 gives HR-TEM images and the FFT-processed lattice point images of In_2_O_3_ cubes before and after annealing. The as-synthesized cube exhibits greater lattice point deviation in the surface layer region, when compared with the inner lattice spots. Significantly, lattice points become highly stretched or even connected throughout the entire cube after annealing. This large degree of lattice distortion from thermal energy can certainly affect light absorption to produce a visible change to the powder color.

**Figure 6:**
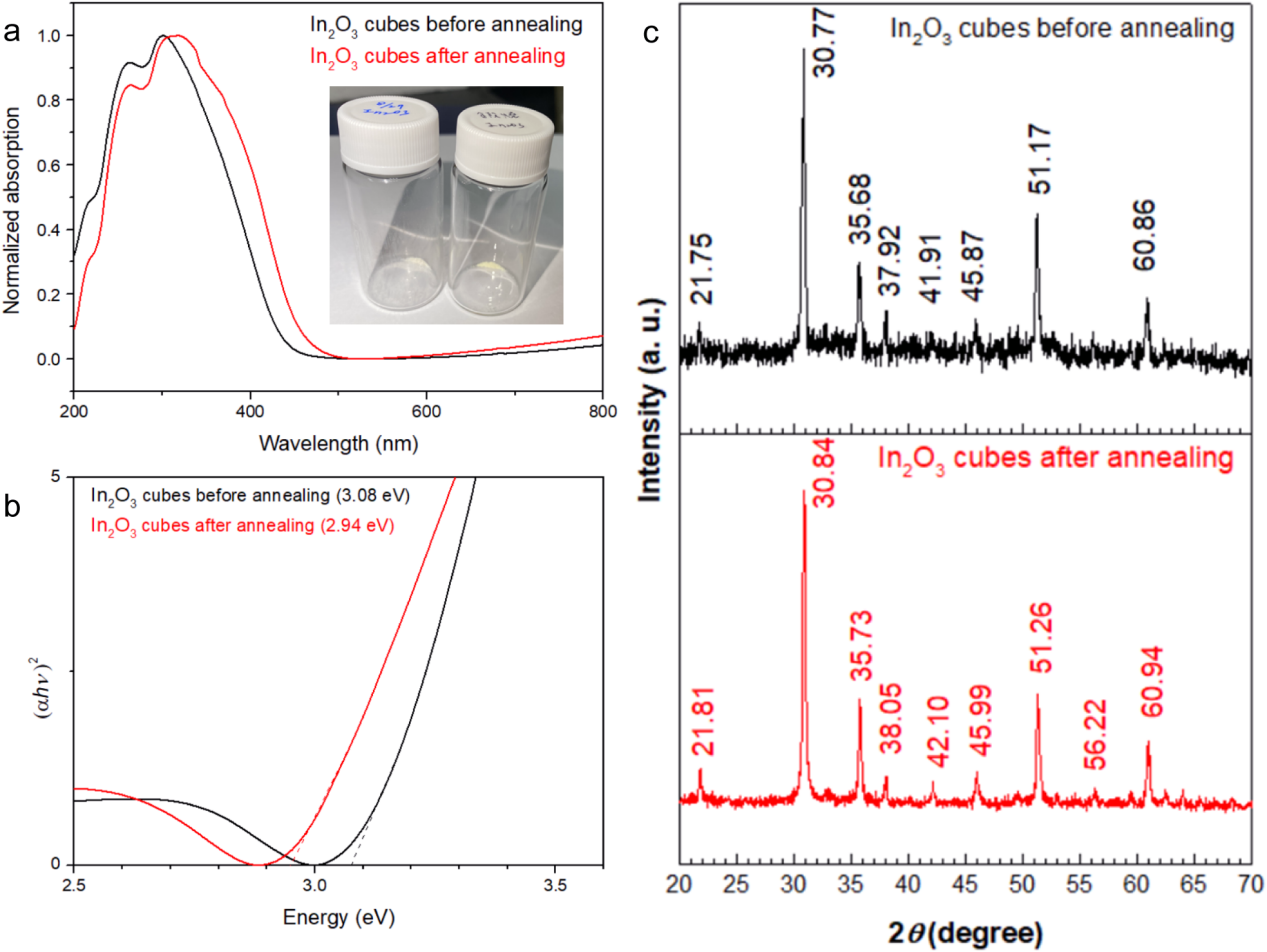
(a, b) Diffuse reflectance spectra and the Tauc plot of another sample of 72 nm In_2_O_3_ cubes before and after annealing to 500 °C for 2 h. Inset gives a photograph of the In_2_O_3_ particles (left) before and (right) after annealing. (c) In-house XRD patterns of the samples showing peak shifts after annealing.

### Photocatalytic Formation of Benzothiazoles

The larger In_2_O_3_ particles can be more easily synthesized, so 72 nm In_2_O_3_ nanocubes were employed for photocatalytic formation of benzothiazole under 370 nm light irradiation with oxygen supplied from a balloon. To determine the optimal reaction conditions, the solvent effect on the photocatalytic synthesis of benzothiazole was first evaluated (Table 1). In aprotic solvents such as acetonitrile and dimethylformamide, the yields were 55% and 56%, respectively. By comparison, reactions carried out in protic solvents including water, methanol/water (1:1), methanol, and ethanol gave yields of 21%, 25%, 82%, and 95%, respectively. The low yields observed in water-containing systems can be attributed to the poor solubility of 2-aminothiophenol and benzaldehyde in water. Ethanol with 95% product yield was chosen for subsequent experiments for its high product yield. These findings indicate that protic solvents are more favorable for the formation of benzothiazole. Since 5% of unreacted benzaldehyde was still detected, the amounts of reagents were reduced by 25% (Table S2). This adjustment successfully drove the reaction to completion, resulting in a quantitative yield of 100%. Figure S8 shows characterization of the particles after the photocatalytic reaction. While the cubic shape is still recognizable, particle fusion appears. XRD pattern indicates preservation of the crystal composition.

**Table 1:**
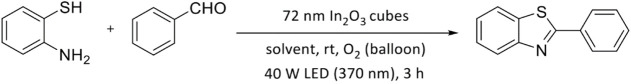
Solvent Effect on the Photocatalytic Synthesis of Benzothiazole

entry^a^	solvent	yield (%)^b^
1	acetonitrile	55
2^c^	dimethylformamide	56
3	water	21
4	methanol/water (1:1)	25
5	methanol	82
6^c^	ethanol	95

a Reagents: In_2_O_3_ nanocubes (2.8 mg), 2-aminothiophenol (0.22 mmol), benzaldehyde (0.2 mmol), solvent (1.6 mL).

b Triphenylmethane as an internal reference.

c The starting material was not completely consumed.

To evaluate the significance of each reaction component, control experiments were conducted (Table 2). In the absence of the In_2_O_3_ catalyst, the reaction afforded a 58% yield, while performing the reaction without light irradiation resulted in a 57% yield. Thus, both the photocatalyst and light irradiation contribute positively to the reaction efficiency. The significant product yield in the absence of In_2_O_3_ crystals suggests that another reaction pathway is operative. This is not unusual; high product yield was also observed in the absence of a solid catalyst in photocatalytic thiophenol coupling with styrene.^31^ Moreover, when the reaction was conducted under air and nitrogen atmosphere, the yields were 54% and 37%, respectively. The results show that molecular oxygen plays a crucial role in the photocatalytic process.

**Table 2:**
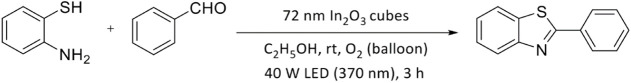
Control Experiments

entry^a^	catalyst	light	O_2_	yield (%)^b^
1	+	+	+	100
2	–	+	+	58
3	+	–	+	57
4	+	+	–^c^	54
5	+	+	–^d^	37

a Reagents: In_2_O_3_ nanocubes (2.8 mg), 2-aminothiophenol (0.165 mmol), benzaldehyde (0.15 mmol), C_2_H_5_OH (1.6 mL).

b Triphenylmethane as an internal reference.

c Air atmosphere.

d N_2_ atmosphere.

A recycling experiment was performed to evaluate the reusability of the In_2_O_3_ catalyst (Figure S9). After the first photocatalytic reaction, the used In_2_O_3_ was recovered by washing once with water and twice with ethanol. The solvent was then removed using a rotary evaporator. The recovered catalyst was weighed to ensure that its mass matched the original 2.8 mg before being reused. In the second cycle, the yield was 87%. The observed partial particle fusion seen in Figure S8 should contribute to the conversion yield decrease. After the second reaction, some catalyst was lost during centrifugation. To eliminate the influence of catalyst mass on the reaction outcome, the catalyst amount was replenished back to 2.8 mg before conducting the third cycle, which delivered a yield of 83%. These results demonstrate that the particles can be reused for multiple catalytic cycles with minimal loss in activity.

Subsequently, substituted benzaldehydes were employed under identical reaction conditions, and the reaction times were adjusted to optimize yields (Table 3). The yield of 2-(4-bromophenyl)­benzothiazole was 69%, that of 2-(4-methylphenyl)­benzothiazole was 71%, and 2-(4-methoxyphenyl)­benzothiazole was obtained at a yield of 64%. Reasonably good yields have been achieved. Proton nuclear magnetic resonance (^1^H NMR) spectra of these products are available in the Supporting Information (Figures S10–S13).

**Table 3:**
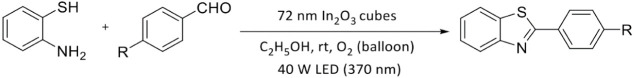

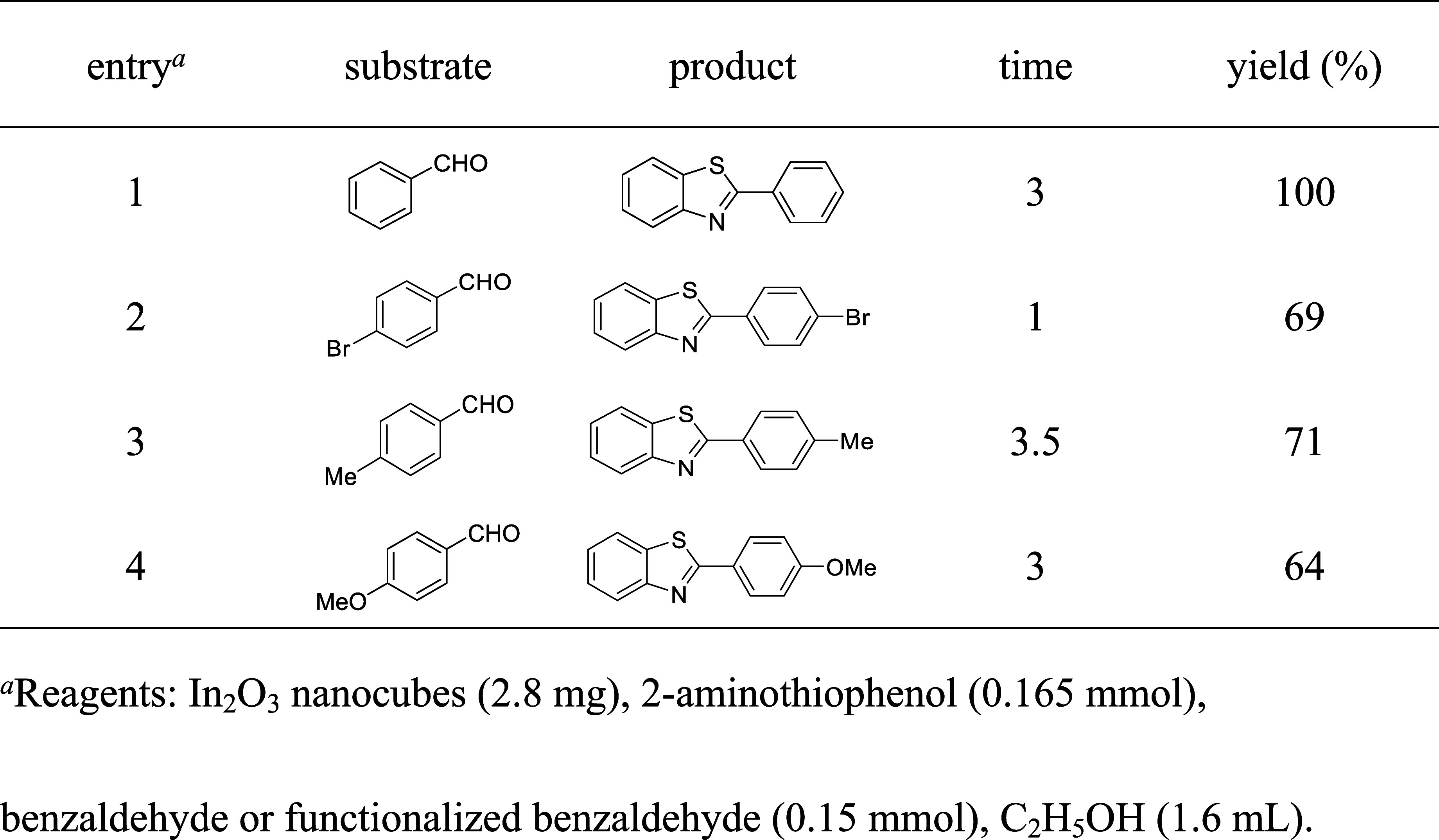
Substrate Scope for Benzothiazole Formation

To gain insight into the reaction mechanism in the synthesis of benzothiazole, a series of active species trapping experiments were conducted (Table 4). In the presence of potassium bromate (KBrO_3_), the yield decreased from 100% to 66%, suggesting the involvement of photogenerated electrons. The use of DIPEA as a hole scavenger led to a significant drop in yield to 34%, implying that holes play an important role in the reaction. When isopropanol was added, the yield remained relatively high at 76%, indicating that hydroxyl radical (^•^OH) is not a key reactive species in this transformation. Furthermore, when DABCO and TEMPO, which quench superoxide radicals (O_2_^•–^) and singlet oxygen (^1^O_2_), were introduced, the yields dropped dramatically to just 17% and 41%, respectively. These results highlight that both superoxide radicals and singlet oxygen are important reactive oxygen species in the photocatalytic mechanism.

**Table 4:**
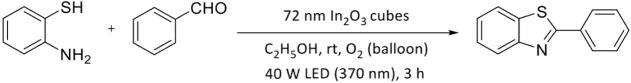
Active Species Trapping Experiments

entry^a^	trapping agent	yield (%)^b^
1	KBrO_3_ (e^–^)	66
2	DIPEA (h^+^)	34
3	isopropanol (^•^OH)	75
4	DABCO (^1^O_2_)	41
5	TEMPO (O_2_^•–^)	17

a Reagents: In_2_O_3_ nanocubes (2.8 mg), 2-aminothiophenol (0.165 mmol), benzaldehyde (0.15 mmol), C_2_H_5_OH (1.6 mL).

b Triphenylmethane as an internal reference.

Electron paramagnetic resonance (EPR) spectroscopy is a powerful tool for identifying active species involved in photocatalytic reactions. DMPO is commonly used as a spin-trapping agent to detect hydroxyl radicals and superoxide anion radicals by forming stable adducts with distinct EPR features.^32^ Figure S14 presents an EPR spectrum of In_2_O_3_ nanocubes under the same 370 nm UV light irradiation, matching more closely to an eight-line signal, which is the signature of the DMPO–OOH adduct. Superoxide radicals are considered the predominant reactive species.

On the basis of the active species trapping experiments and the EPR spectral result, a mechanism for the photocatalytic formation of benzothiazole is proposed (Figure 7). Initially, benzaldehyde reacts with 2-aminothiophenol through a condensation reaction to form intermediate I, accompanied by the elimination of water. This intermediate then undergoes intramolecular cyclization to generate intermediate II. Upon UV light irradiation on In_2_O_3_ nanocubes, excited electrons and holes are produced. The photogenerated holes oxidize intermediate II to form intermediate III, while the excited electrons are transferred to molecular oxygen to give superoxide radicals. These radicals then deprotonate intermediate III, generating intermediate IV. The resulting hydroperoxyl radicals (HOO^•^) abstract a hydrogen atom from intermediate IV, yielding benzothiazole along with hydrogen peroxide as a byproduct. Alternatively, a secondary pathway may involve the photoexcited electrons relaxing to a lower energy level, releasing energy that activates molecular oxygen into singlet oxygen (^1^O_2_). Light can also directly energize molecular oxygen to form singlet oxygen, so this is the pathway in the absence of In_2_O_3_ crystals. The singlet oxygen oxidizes intermediate II to form intermediate V, which subsequently undergoes H elimination to yield benzothiazole and H_2_O_2_.^27,33^ The more dramatic product yield decrease in the presence of a superoxide radical scavenger compared to that for the singlet oxygen scavenger suggests that the In_2_O_3_ nanocubes contribute positively to photocatalytic efficiency.

**Figure 7:**
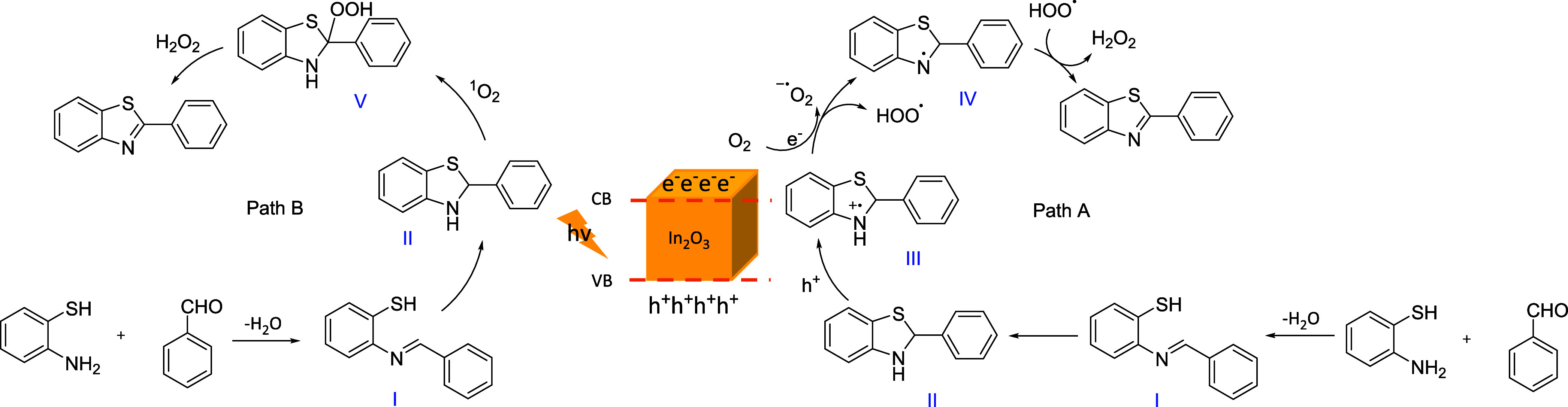
Proposed mechanism for photocatalytic benzothiazole synthesis.

## Conclusions

In_2_O_3_ nanocubes with tunable sizes of 10, 72, and 95 nm have been solvothermally synthesized in ethanol. Synchrotron XRD analysis shows symmetric peaks for 10 nm cubes, but those of larger cubes can be deconvoluted into bulk and surface layer components. From FFT-processed HR-TEM images, lattice points in the surface layer region present greater deviations than those in the crystal interior for the 72 and 95 nm cubes, but more uniform spot feature throughout the interior of the 10 nm cube. These results provide structural variation evidence and explain the observation of optical size effect in semiconductor nanocrystals. Indeed, band gaps of 10 and 95 nm cubes differ by 0.13 eV. By heating the 72 nm nanocubes to high temperatures, the white crystals become yellowish from significant lattice distortion, demonstrating that the observed semiconductor color variation can also link to lattice differences. The In_2_O_3_ nanocubes were applied to photocatalyze the formation of benzothiazole by coupling 2-aminothiophenol and benzaldehyde, achieving an excellent yield of 100%. Substituted benzothiazoles have also been synthesized. Superoxide radicals and singlet oxygen are active species in the organic transformation. More examples of detailed lattice examination should establish the origin of size-dependent optical behaviors of semiconductor nanocrystals.

## Experimental Section

### Synthesis of Size-Controlled In_2_O_3_ Nanocubes

Initially, 0.2 M In­(NO_3_)_3_, 0.03 M NaOH, and 0.3 M NaOH solutions in ethanol were prepared. Teflon vessels were used to synthesize 10 nm In_2_O_3_ nanoparticles, while glass tubes from Tung Kuang Glass were employed for growing 72 and 95 nm particles. A much longer reaction time was needed to obtain 10 nm cubes, so a Teflon container was chosen for its superior heat resistance. In contrast, 72 and 95 nm cubes were synthesized in just 2 h, allowing the use of glass pressure tubes. The changes were found necessary to ensure formation of individual nanoparticles. The detailed reaction conditions and reagent quantities are summarized in Table S3. In a typical procedure, the In­(NO_3_)_3_ solution was added to specific volumes of ethanol and stirred for 10 min. Subsequently, the NaOH solution was added dropwise, followed by 10 min of stirring to ensure homogeneity. A slight whitening of the solution was observed. The resulting suspension was transferred to an oven and heated at 180 °C for 20 h to make 10 nm cubes. To synthesize 72 and 95 nm cubes, the solutions were heated at 160 and 180 °C for just 2 h, respectively. Figure S15 illustrates the synthesis procedure. After the reaction, the mixtures were cooled to room temperature. The resulting white precipitates were collected by centrifugation at 10,000 rpm for 1.5 min, and washed several times with distilled water and ethanol to remove residual ions and impurities. No uncommon hazards are noted.

### Photocatalytic Formation of Benzothiazoles

To synthesize benzothiazoles, 2.8 mg of 72 nm In_2_O_3_ cubes were first placed into a 15 mL oven-dried quartz tube, which was then sealed with a rubber septum. Air inside the quartz tube was removed using a vacuum system and subsequently replaced with an oxygen-filled balloon. This evacuation–refilling process was repeated three times to ensure the tube was fully saturated with oxygen. Next, 0.165 mmol of 2-aminothiophenol and 0.15 mmol of benzaldehyde were dissolved in 1.6 mL of solvent. The resulting solution was injected into the oxygen-purged quartz tube containing the catalyst using a syringe. After thorough mixing by sonication, the reaction mixture was stirred and irradiated with a 40 W LED (λ = 370 nm) for 3 h. A fan was used to dissipate heat during the reaction to prevent thermal catalysis (Figure S16). Upon completion, the reaction mixture was centrifuged at 11,000 rpm for 3 min to separate the catalyst. The supernatant was then concentrated under rotary evaporator to remove the solvent, affording the desired product.

### Instrumentation

XRD patterns were obtained using a Bruker D2 PHASER diffractometer with Cu *K*α radiation. Synchrotron XRD measurements were carried out at beamline 19A of the Taiwan Photon Source (TPS 19A), National Synchrotron Radiation Research Center. A high-brilliance hard X-ray beam was generated using an in-vacuum cryogenic undulator (CU15), and the powder diffraction data were collected using a MYTHEN 18K position-sensitive detector. For the 10 and 72 nm In_2_O_3_ samples, diffraction data were obtained by averaging ten measurements, each with a 30 s exposure. For the 95 nm In_2_O_3_ sample, a 10 s single exposure was used to avoid thermal interference caused by prolonged irradiation. SEM images were collected using a JEOL JSM-70000F electron microscope. UV–vis diffuse reflectance spectra were taken using a JASCO V-670 spectrometer with a solid sample holder attached. HR-TEM characterization was performed using a JEOL JEM-2100 electron microscope for 10 nm cubes and a JEOL JEM-ARM200FTH electron microscope for larger cubes. A Bruker ELEXSYS E-580 spectrometer was used to obtain electron paramagnetic resonance spectra. ^1^H NMR spectra were recorded on a Bruker Avance II 400 MHz spectrometer at room temperature (25 °C). Chemical shifts (δ) are given in parts per million (ppm) relative to the residual solvent peak of *d*-DMSO (δ = 2.50 ppm) as an internal standard.

## Supplementary Material



## Notes

Additional experimental details, crystal synthesis conditions, size distribution histograms, XRD patterns, Rietveld refinement results, TEM images, SEM images, graph of product yields using recycled particles, EPR spectrum, and NMR data (PDF)

## References

[1] Lee A.-T., Tan C.-S., Huang M. H. (2021). Current Rectification and Photo-Responsive
Current Achieved through Interfacial Facet Control of Cu _2_ O–Si Wafer Heterojunctions. ACS Cent.
Sci..

[2] Dai W.-T., Wen C.-C., Lin H.-J., Huang M. H. (2025). Photocatalyzed Aerobic
Oxidation of Thiols to Disulfides Using Cu_2_O Polyhedra. ACS Appl. Mater. Interfaces.

[3] Chang P.-S., Chen B.-H., Lin Y.-C., Dai W.-T., Kumar G., Lin Y.-G., Huang M. H. (2024). Growth of Size-Tunable
Ag_2_O Polyhedra and Revelation of Their Bulk and Surface
Lattices. Small.

[4] Adamowicz W., Macky W., Kobielusz M. (2025). Facet-Dependent Photocatalytic Reduction
of Nitroaromatics Using Tailored SrTiO_3_ Crystals: Mechanism
and Reactivity Enhancement. J. Mater. Chem.
A.

[5] Hsieh P.-L., Naresh G., Huang Y.-S., Tsao C.-W., Hsu Y.-J., Chen L.-J., Huang M. H. (2019). Shape-Tunable
SrTiO_3_ Crystals
Revealing Facet-Dependent Optical and Photocatalytic Properties. J. Phys. Chem. C.

[6] Chuang Y.-J., Pal A., Chen B.-H., Jena S., Suresh S., Lin Z.-H., Huang M. H. (2025). Synthesis of Shape-Tunable
PbZrTiO_3_ Nanocrystals
with Lattice Variations for Piezoelectric Energy Harvesting and Human
Motion Detection. Chem. Sci..

[7] Yang Y.-T., Chen B.-H., Pal A., Li C.-H., Lin Z.-H., Huang M. H. (2025). Lattice Variations in CaTiO_3_ Cubes and Cuboids
and Their Use in Photocatalytic Benzimidazole Formation. J. Mater. Chem. A.

[8] Kumar G., Sun H.-W., Huang M. H. (2024). Shape-Tunable Co_3_O_4_ Nanocrystals Possessing Surface-Dependent Optical and Magnetic
Properties. ACS Appl. Nano Mater..

[9] Yang J.-H., Wang C.-P., Chen B.-H., Huang M. H. (2025). Growth of Fe_3_O_4_ Truncated Cubes
and Rhombic Dodecahedra Showing
Interior Lattice and Magnetic Behavior Variations. Inorg. Chem..

[10] Pal A., Chen B.-C., Dai W.-T., Yang C.-C., Lin Z.-H., Wu H.-J., Tan C.-S., Huang M. H. (2025). Silicon Wafers Exhibiting
Highly Surface-Related Thermoelectric Properties. J. Phys. Chem. C.

[11] Chen B.-H., Kumar G., Wei Y.-J., Ma H.-H., Kao J.-C., Chou P.-J., Chuang Y.-C., Chen I.-C., Chou J.-P., Lo Y.-C. (2023). Experimental Revelation
of Surface and Bulk Lattices
in Faceted Cu_2_O Crystals. Small.

[12] Huang J.-Y., Madasu M., Huang M. H. (2018). Modified Semiconductor
Band Diagrams
Constructed from Optical Characterization of Size-Tunable Cu_2_O Cubes, Octahedra, and Rhombic Dodecahedra. J. Phys. Chem. C.

[13] Hsiao C.-H., Chen C.-W., Chen H.-S., Hsieh P.-L., Chen Y.-A., Huang M. H. (2021). Formation of Size-Tunable CdS Rhombic
Dodecahedra. J. Mater. Chem. C.

[14] Ma H.-H., Huang M. H. (2023). Size- and Facet-Dependent
Photoelectrochemical Properties
of Cu_2_O Crystals. J. Mater. Chem.
C.

[15] Yang Z.-L., Kumar G., Huang M. H. (2022). Synthesis
of Zinc Blende-Phased CdSe
Nanocrystals with Size-Tunable Optical Properties and Adjustable Valence
Band Positions. Langmuir.

[16] Kumar G., Chen C.-R., Chen B.-H., Chen J.-W., Huang M. H. (2022). Morphological
Evolution of Cadmium Oxide Crystals Showing Color Changes and Facet-Dependent
Conductivity Behavior. J. Mater. Chem. C.

[17] Chen B.-W., Chen B.-H., Yang P.-Y., Jena S., Yang S.-J., Hsu Y.-J., Huang M. H. (2025). Size-Tunable
BaZrO_3_ Cubes
and Rhombic Dodecahedra Exhibiting Facet-Dependent Optical, Photocatalytic,
and Piezoelectric Behaviors. Chem.–Asian
J..

[18] Chen C.-K., Chen B.-H., Huang M. H. (2023). Low-Temperature Growth of Rock Salt
MnS Nanocrystals with Facet-Dependent Behaviors. Chem. Mater..

[19] Huang Y., Mao X., Yuan G., Zhang D., Pan B., Deng J., Shi Y., Han N., Li C., Zhang L. (2021). Size-Dependent
Selectivity of Electrochemical CO_2_ Reduction on Converted
In_2_O_3_ Nanocrystals. Angew.
Chem., Int. Ed..

[20] Meng M., Wu X., Zhu X., Yang L., Gan Z., Zhu X., Liu L., Chu P. K. (2014). Cubic In_2_O_3_ Microparticles for
Efficient Photoelectrochemical Oxygen Evolution. J. Phys. Chem. Lett..

[21] Yang J., Li C., Quan Z., Kong D., Zhang X., Yang P., Lin J. (2008). One-Step Aqueous Solvothermal Synthesis of In_2_O_3_ Nanocrystals. Cryst. Growth Des..

[22] Suzuki T., Watanabe H., Ueno T., Oaki Y., Imai H. (2017). Significant
Increase in Band Gap and Emission Efficiency of In_2_O_3_ Quantum Dots by Size-Tuning around 1 nm in Supermicroporous
Silicas. Langmuir.

[23] Buchholz D. B., Ma Q., Alducin D., Ponce A., Jose-Yacaman M., Khanal R., Medvedeva J. E., Chang R. P. H. (2014). The Structure
and Properties of Amorphous Indium Oxide. Chem.
Mater..

[24] Shao M., Chen H., Shen M., Chen W. (2017). Synthesis and Photocatalytic
Properties of In_2_O_3_ Micro/Nanostructures with
Different Morphologies. Coll. Surf. A.

[25] Sethiya A., Sahiba N., Teli P., Soni J., Agarwal S. (2022). Current Advances
in the Synthetic Strategies of 2-Arylbenzothiazole. Mol. Divers.

[26] Gao X., Liu J., Zuo X., Feng X., Gao Y. (2020). Recent Advances
in
Synthesis of Benzothiazole Compounds Related to Green Chemistry. Molecules.

[27] Feng J., Yang B., Fan P., Lu Y., Su Q., Li B., Yang Q., Lei M., Ren H., Wu Q. (2025). Hydrazone-Based
Covalent Organic Frameworks for Efficient Photocatalytic Redox Reactions. J. Mater. Chem. A.

[28] Jiang X., Su J., Ma W., Qiu B., Li Y., Xia X., Kang X., Zhang J., Zhou H. (2025). Modulation of π-Conjugation
Length in Vinylene-Linked Covalent Organic Frameworks for Efficient
Photocatalysis. ACS Mater. Lett..

[29] Chang S.-C., Huang M. H. (2008). Formation of Short
In_2_O_3_ Nanorod
Arrays within Mesoporous Silica. J. Phys. Chem.
C.

[30] Jena S., Hsieh M.-C., Huang M. H. (2025). Formation
of ZnS Cubes and Quantum
Dots and Structural Feature for Improved Quantum Dot Emission. Langmuir.

[31] Su Y.-S., Chen B.-H., Huang M. H. (2026). Size-Tunable Zn_2_SnO_4_ Octahedra Possessing Two Lattice Phases for
the Thiol–Ene
Reaction. J. Mater. Chem. A.

[32] Ma J., Wang C., He H. (2016). Enhanced Photocatalytic Oxidation
of NO over g-C_3_N_4_-TiO_2_ under UV and
Visible Light. Appl. Catal., B.

[33] Shao J., Wang H., Tao X., Zhu G. (2025). Optimal Photosynthesis
of 2-Benzothiazoles over Hexaazatrinaphthylene-Based Porous Aromatic
Frameworks. Chem. Sci..

