# Supplementary material for: Structural Origin of Semiconductor Optical Size Effect through In2O3 Nanocubes and Use in Photocatalytic Benzothiazole Formation
Source: Inorg Chem. 2026 May 28;65(22):12619–28. doi: 10.1021/acs.inorgchem.6c01581 (PMC13250991; doi:10.1021/acs.inorgchem.6c01581)
Supplement: Supplementary file 1 [file ic6c01581_si_001.pdf]

Supporting Information

# Structural Origin of Semiconductor Optical Size Effect through $In_2O_3$ Nanocubes and Use in Photocatalytic Benzothiazole Formation

Xin-Ru Lin,† Kuo-Chang Chien,† Bo-Hao Chen,†‡ and Michael H. Huang*†
†*Department of Chemistry, National Tsing Hua University, Hsinchu 300044, Taiwan*
‡*National Synchrotron Radiation Research Center, Hsinchu 300092, Taiwan*
E-mail: hyhuang@mx.nthu.edu.tw

**Chemicals.** Indium nitrate hydrate ($In(NO_3)_3$, 99.9%, Thermo Scientific), sodium hydroxide (NaOH, 98%, Honeywell), ethanol ($C_2H_5OH$, 99.8%, Honeywell), methanol ($CH_3OH$, 99.8%, Honeywell), 2-propanol ($(CH_3)_2CHOH$, 99.9%, Echo), acetonitrile ($CH_3CN$, 99.5%, J. T. Baker), dimethylformamide (DMF, $C_3H_7NO$, 99.5%, Merck) benzaldehyde ($C_6H_5CHO$, 98%, Sigma), 2-aminothiophenol ($C_6H_4(SH)(NH_2)$, 98%, Thermo Scientific), triphenylmethane ($(C_6H_5)_3CH$, 98%, NOVA), TEMPO ($C_9H_{18}NO$, 98%, Alfa Aesar), DABCO ($C_6H_{12}N_2$, 98%, Alfa Aesar), DMPO ($C_6H_{11}NO$, 98%, Matrix Scientific), potassium bromate ($KBrO_3$, 99%, Alfa Aesar), N,N-diisopropylethylamine ($C_8H_{19}N$, 99.5%, Acros Organics), 4-bromobenzaldehyde ($C_7H_5BrO$, NOVA, 99%), and p-tolualdehyde ($C_8H_8O$, 98%, NOVA) were used in this study.

**Active Species Scavenging Experiments.** In this experiment, 2.8 mg of 72 nm $In_2O_3$ nanocubes was placed in a 15 mL dried quartz tube and sealed with a rubber septum. The tube was evacuated using a vacuum system and backfilled with an oxygen-filled balloon. This evacuation–refilling cycle was repeated three times to ensure an oxygen-rich environment. Benzaldehyde (0.15 mmol), 2-aminothiophenol (0.165 mmol), and a scavenger (0.15 mmol) were dissolved in 1.6 mL of ethanol and injected into the tube using a syringe. After thorough mixing by sonication, the reaction mixture was stirred and irradiated with a 40 W LED ($\lambda$ = 370 nm) for 3 h. Subsequent procedures follow the standard reaction conditions.

**Electron Paramagnetic Resonance Experiment.** Since commercially available DMPO (5,5-dimethyl-1-pyrroline-N-oxide) often contains impurities, purification with activated carbon is necessary before use. Specifically, 30 mg of DMPO was dissolved in 6 mL of methanol and treated with activated carbon. The mixture was sonicated for 2 min to enhance impurity adsorption. Afterward, centrifugation was performed to remove the activated carbon, yielding a purified DMPO solution, which was then diluted with ethanol with a 1:1 volume ratio.

Under ambient conditions, 2.8 mg of 72 nm $In_2O_3$ cubes was mixed with the DMPO solution in a 15 mL quartz tube that had been oven-dried. The mixture was first sonicated for 1 min, then irradiated with visible light using a 370 nm LED for 2 min. After irradiation, the sample was immediately transferred to a glass vial,

wrapped in aluminum foil to prevent light exposure, and promptly sent to the NTHU EPR lab for analysis.

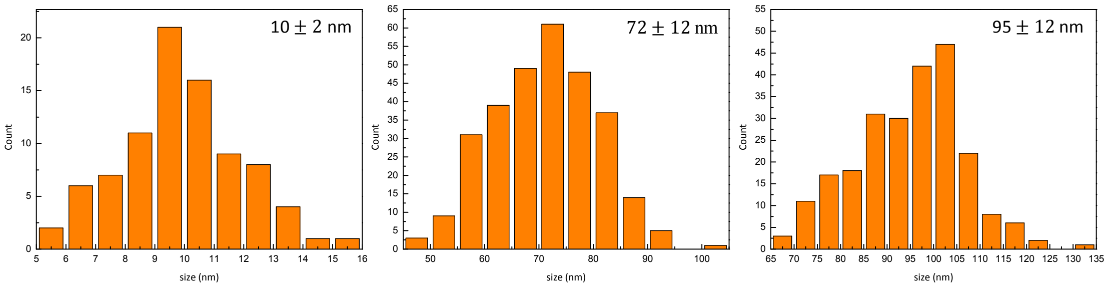


**Figure S1.** Size distribution histograms of the synthesized $In_2O_3$ nanocubes.

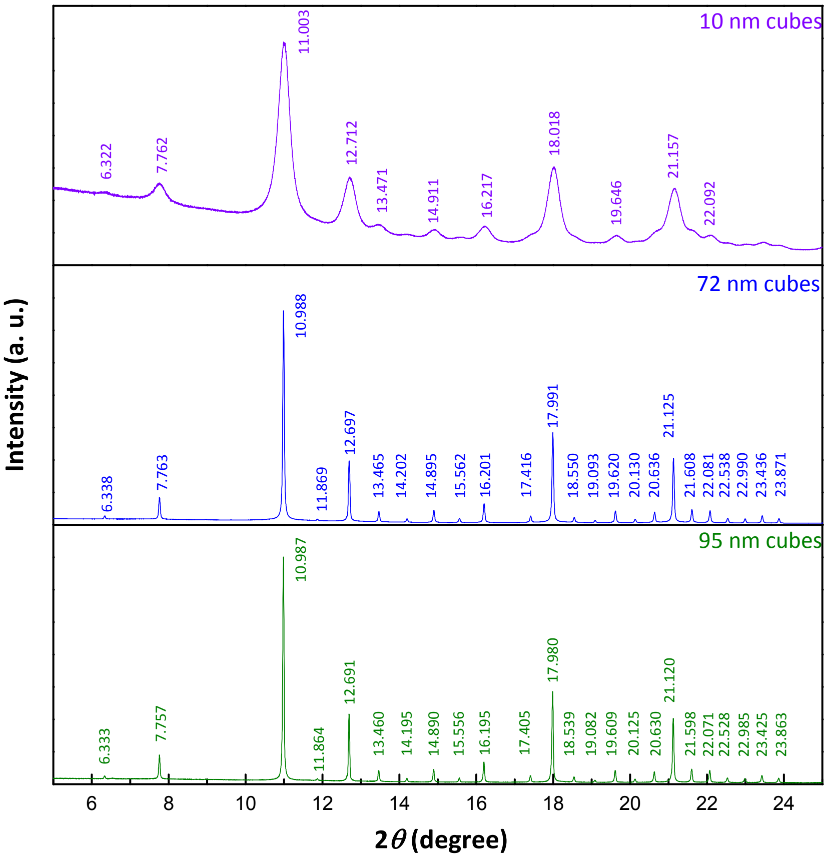


**Figure S2.** Synchrotron XRD patterns of the $In_2O_3$ nanocubes.

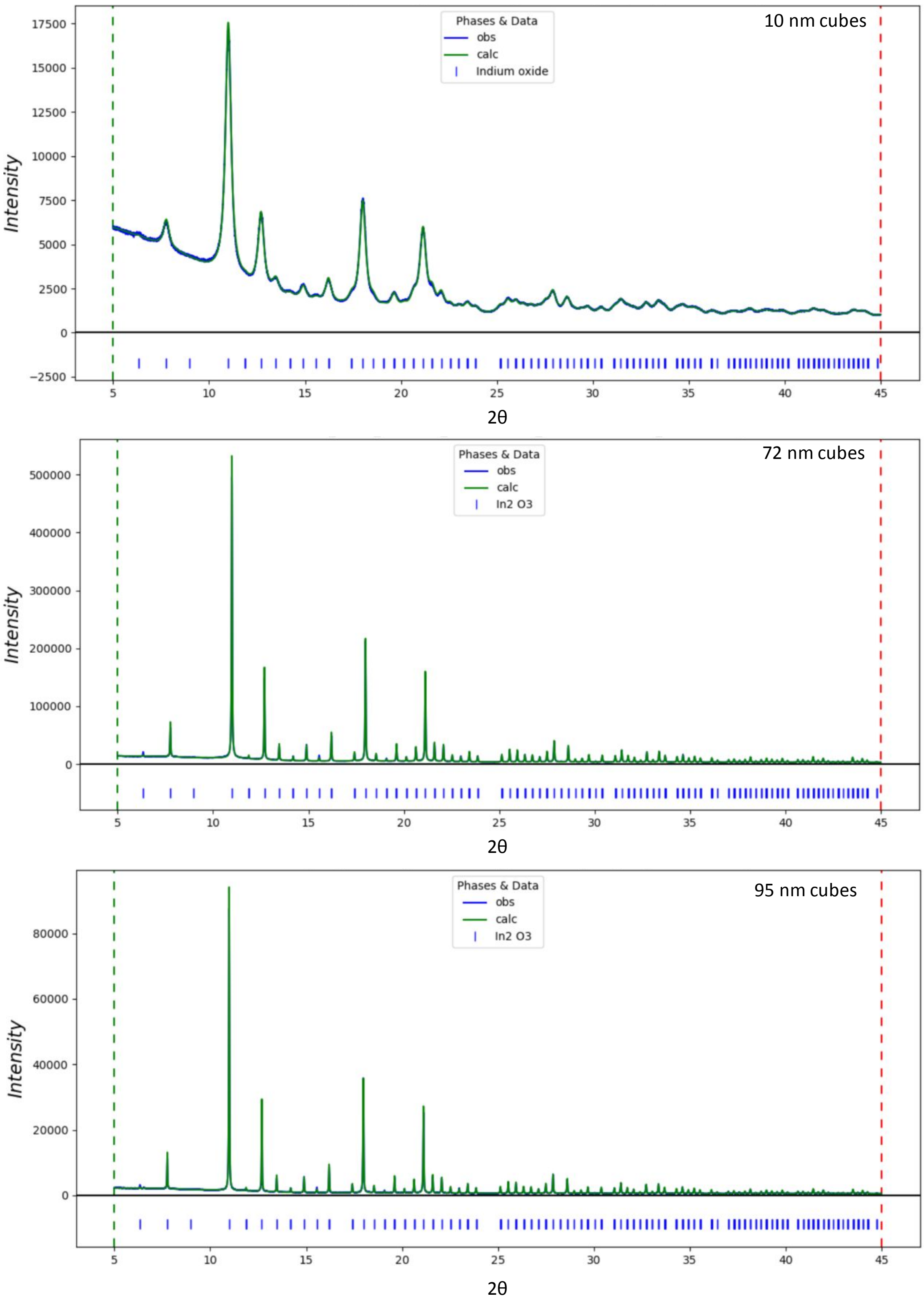


**Figure S3.** Rietveld refinement of the synchrotron XRD patterns of 10, 72, and 95 nm $In_2O_3$ nanocubes.

## Table S1. Rietveld Refinement Parameters of $In_2O_3$ Nanocubes

| formula | $In_2O_3$ | | | | |
|---|---|---|---|---|---|
| FW (g/mol) | 555.27 | | | | |
| space group | I a -3 | | | | |
| λ (Å) | 0.56025 | | | | |
| morphology | cubes | | | | |
| size (nm) | 10 | 72 | | 95 | |
| | | bulk | surface | bulk | surface |
| weight percentage (%) | | 92.2 (3) | 7.8 (3) | 89.79(24) | 10.21(24) |
| unit cell *a* (Å) | 10.12438(22) | 10.13510(4) | 10.12307(12) | 10.13820(4) | 10.12782(10) |
| μ strain unique axis (*hkl*) | 111 | 100 | 111 | 100 | 111 |
| equatorial μ strain $\Delta d/d$ (× $10^6$) | 5306.2 | 1767.9 | 1426.5 | 2370.9 | 1418.4 |
| axial μ strain $\Delta d/d$ (× $10^6$) | 8723.4 | 1533.3 | 2980.2 | 1766.1 | 2699.9 |
| $RF^2$ (%) | 0.856 | 2.57 | 3.40 | 1.83 | 3.561 |
| *wR*p (%) | 1.912 | 4.677 | | 6.150 | |
| zero shift (°) | -0.00015 | 0.00176 | | 0.00305 | |
| 2ϑ range (°) | 5.0 to 45 | 5.0 to 45 | | 5.0 to 45 | |
| *d* resolution (Å) | 0.734 | 0.734 | | 0.735 | |

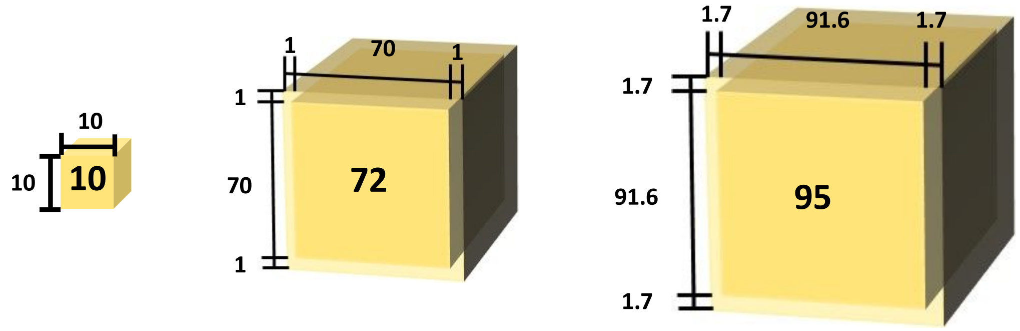


**Figure S4.** Estimated surface layer thicknesses of $In_2O_3$ nanocubes, derived from Rietveld refinement.

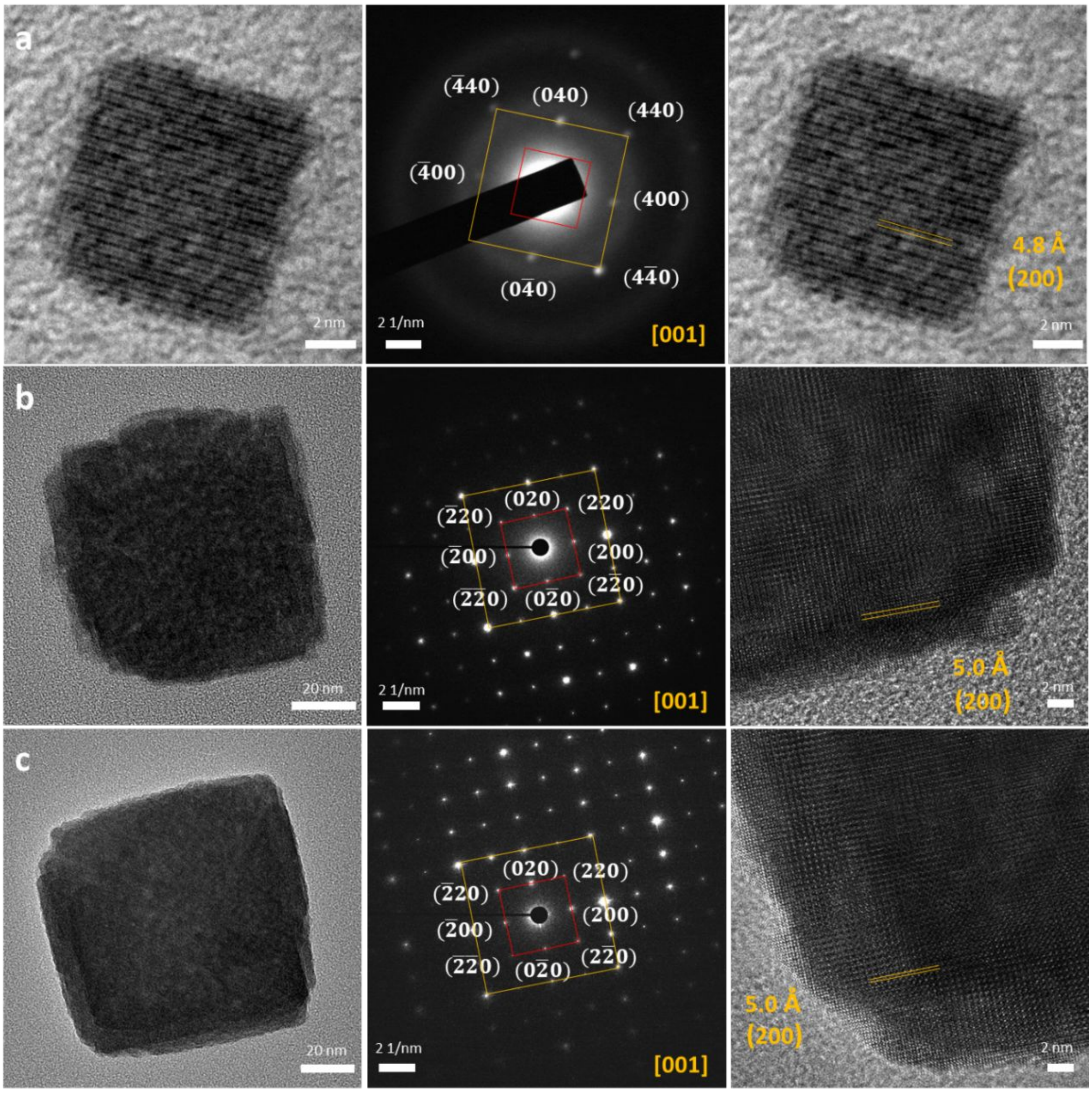


**Figure S5.** TEM images, SAED patterns, and HR-TEM images of (a) 10, (b) 72, and (c) 95 nm $In_2O_3$ cubes.

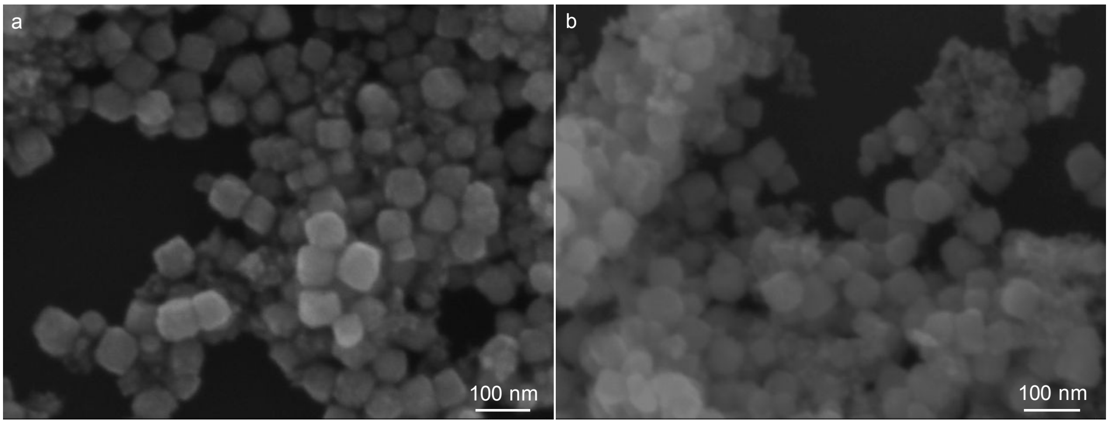


**Figure S6.** SEM images of the 72 nm $In_2O_3$ cube sample (a) before and (b) after annealing.

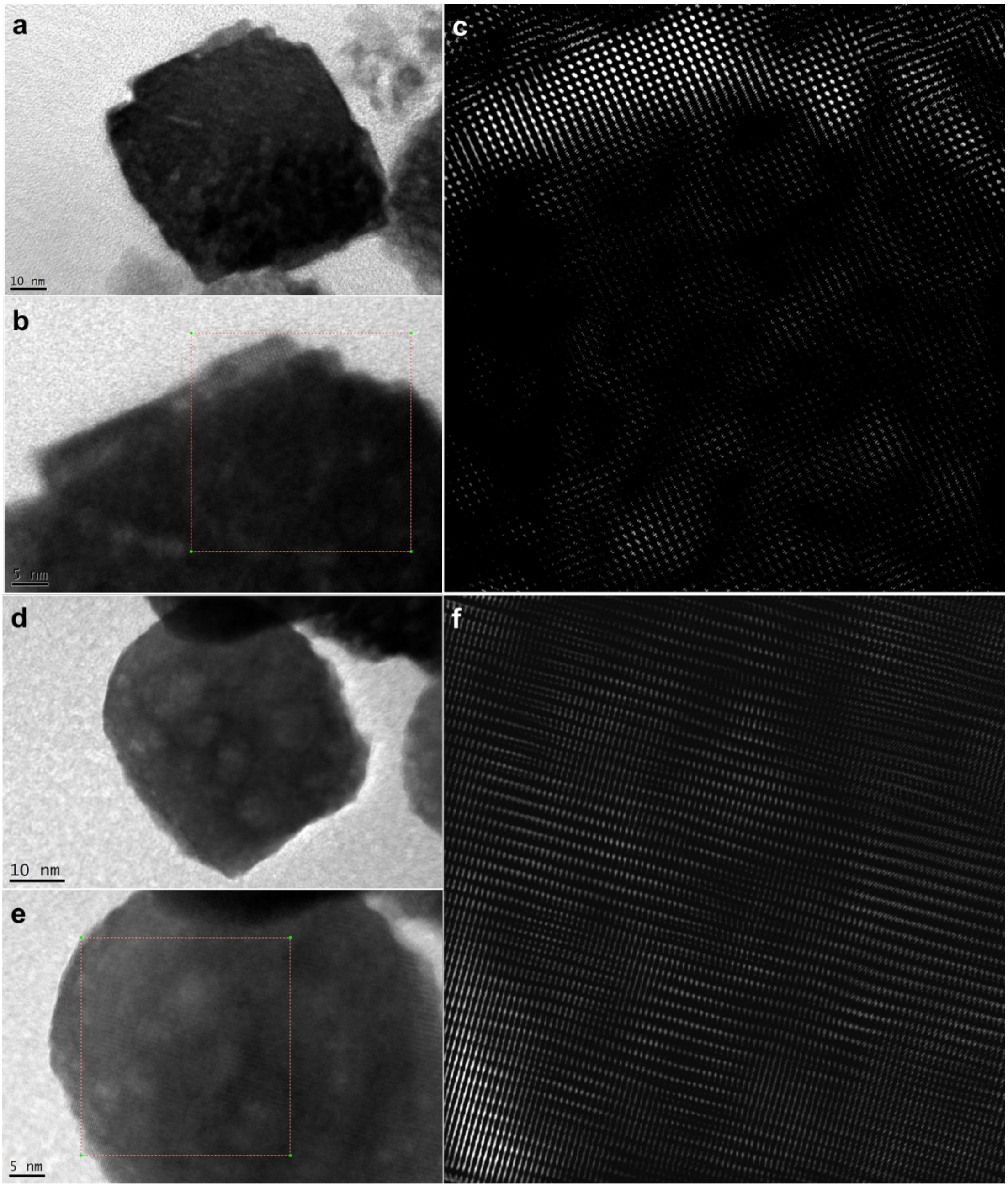


**Figure S7.** (a) TEM and (b) HR-TEM images of a single $In_2O_3$ nanocube before annealing. (c) FFT lattice point image of the framed region in panel b. (d–f) TEM and HR-TEM images of a single $In_2O_3$ nanocube after annealing, and the FFT lattice point image of the framed region in panel e.

**Table S2. Effect of Reagent Amount on the Photocatalytic Activity**

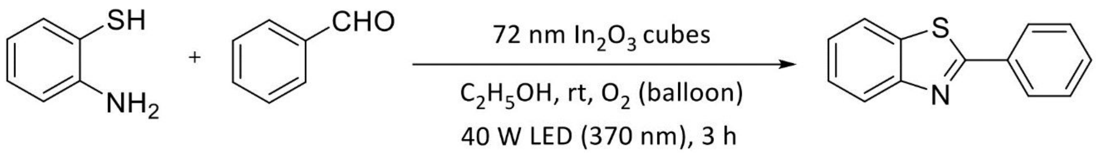


| entry | benzaldehyde (mmol) | 2-aminothiophenol (mmol) | yield (%) |
|---|---|---|---|
| 1 | 0.15 | 0.165 | 100 |
| 2 | 0.2 | 0.22 | 95 |

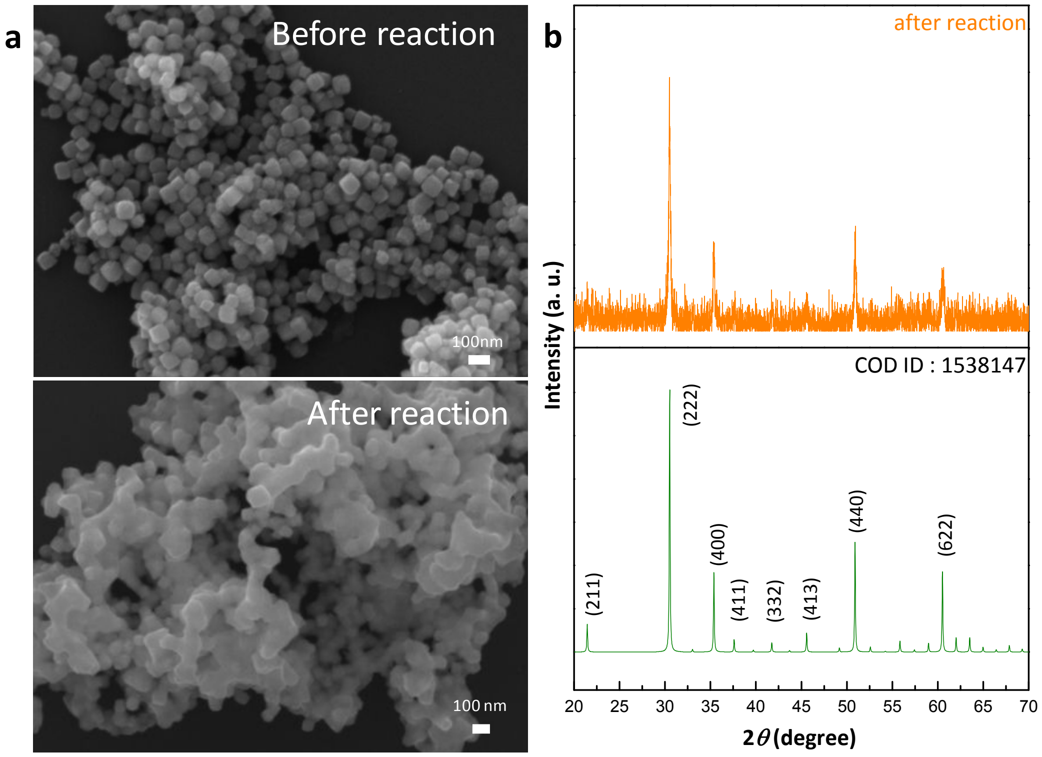


**Figure S8.** (a) SEM images of $In_2O_3$ nanocubes before and after the photocatalytic reaction. (b) XRD pattern of $In_2O_3$ nanocubes after the photocatalytic reaction. A reference pattern is provided.

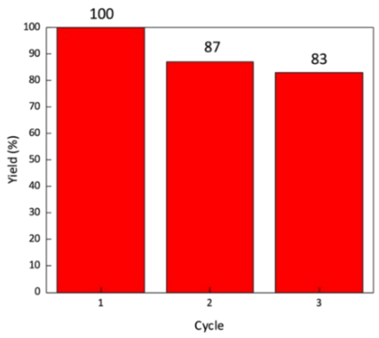


**Figure S9.** Benzothiazole yields in the recycling experiments.

**Spectroscopic Data**

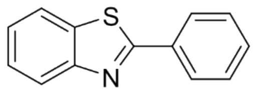


**2-Phenylbenzothiazole (1)**

$^1$H NMR (400 MHz, $CDCl_3$): δ 8.09-8.07 (m, 3H), 7.89-7.87 (d, $J$ = 8.0 Hz, 1H), 7.48-7.47 (m, $J$ = 8.0 Hz, 4H), 7.39-7.35 (t, $J$ = 8.0 Hz, 1H).

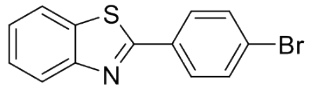


**2-(4-bromophenyl)benzothiazole (2)**

$^1$H NMR (400 MHz, $CDCl_3$): δ 8.06-8.04 (d, $J$ = 8.0 Hz, 1H), 7.93-7.91 (d, $J$ = 8.0 Hz, 2H), 7.88-7.86 (d, $J$ = 8.0 Hz, 1H), 7.60-7.59 (d, $J$ = 4.0 Hz, 2H), 7.50-7.46 (t, $J$ = 8.0 Hz, 1H), 7.39-7.36 (t, $J$ = 8.0 Hz, 1H).

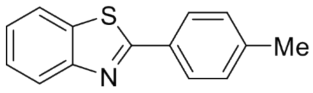


**2-(4-methylphenyl)benzothiazole (3)**

$^1$H NMR (400 MHz, $CDCl_3$): δ 8.07-8.05 (d, $J$ = 8.0 Hz, 1H), 7.98-7.96 (d, $J$ = 8.0 Hz, 2H), 7.87-7.85 (d, 1H), 7.49-7.45 (t, $J$ = 8.0 Hz,1H), 7.37-7.33 (t, $J$ = 8.0 Hz, 1H),7.28-7.26 (d, $J$ = 8.0 Hz, 2H), 2.40 (s, 3H, $CH_3$).

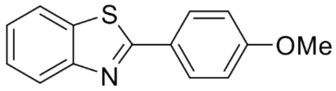


**2-(4-methoxyphenyl)benzothiazole (4)**

$^1$H NMR (400 MHz, $CDCl_3$): δ 8.03–8.00 (m, 3H), 7.82-7.80 (d, $J$ = 8.0 Hz, 1H), 7.48-7.44 (t, $J$ = 8.0 Hz, 1H), 7.36-7.32 (t, $J$ = 8.0 Hz, 1H), 6.98-6.96 (d, $J$ = 8.0 Hz, 2H), 3.87 (s, 3H, $OCH_3$).

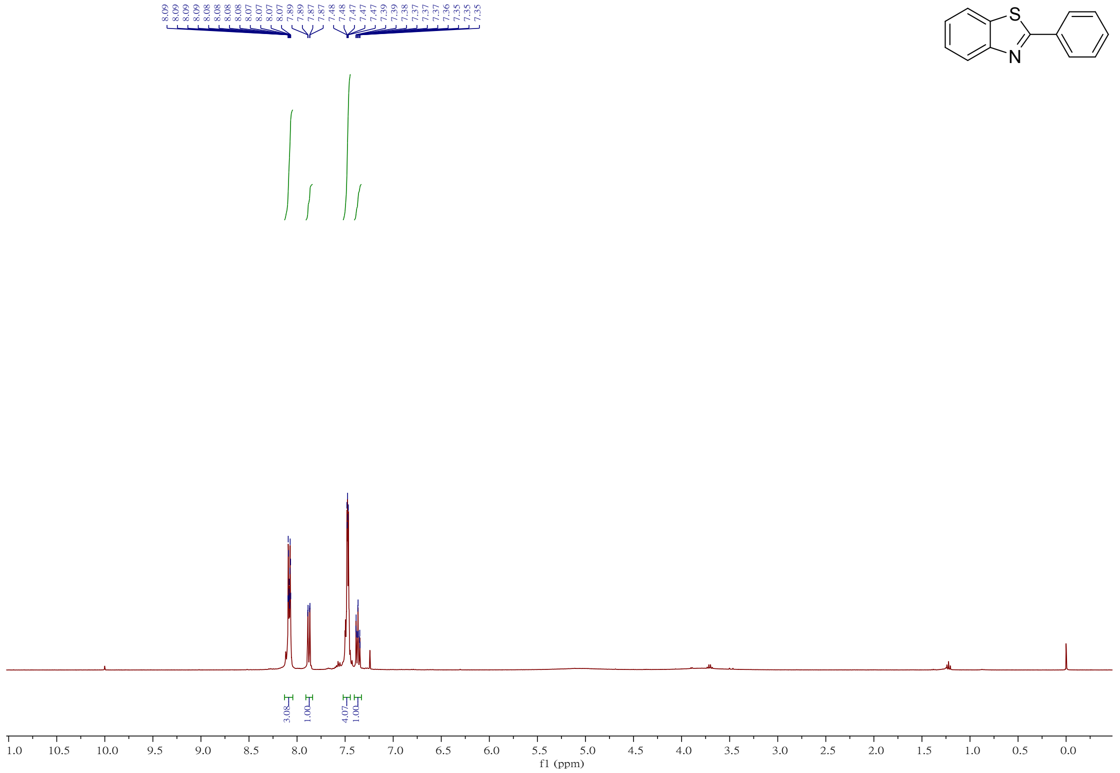


**Figure S10.** Crude $^{1}H$ NMR spectrum of compound 1 ($CDCl_3$, 400 MHz).

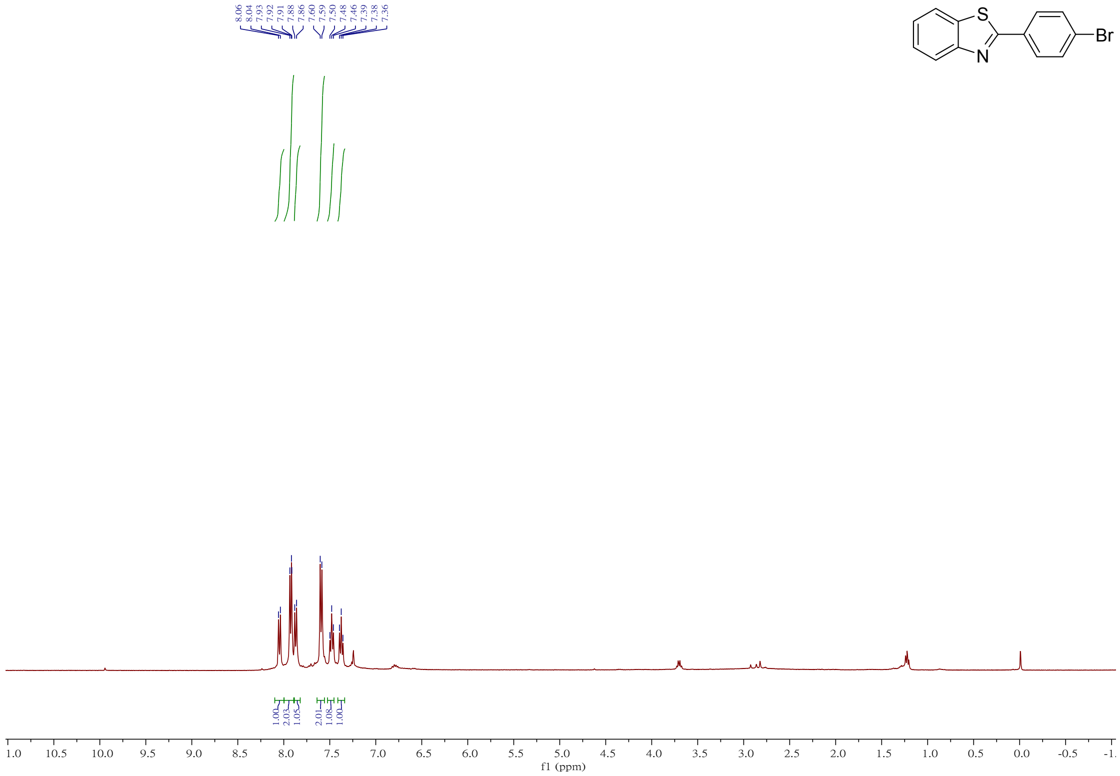


**Figure S11.** Crude $^{1}H$ NMR spectrum of compound 2 ($CDCl_3$, 400 MHz).

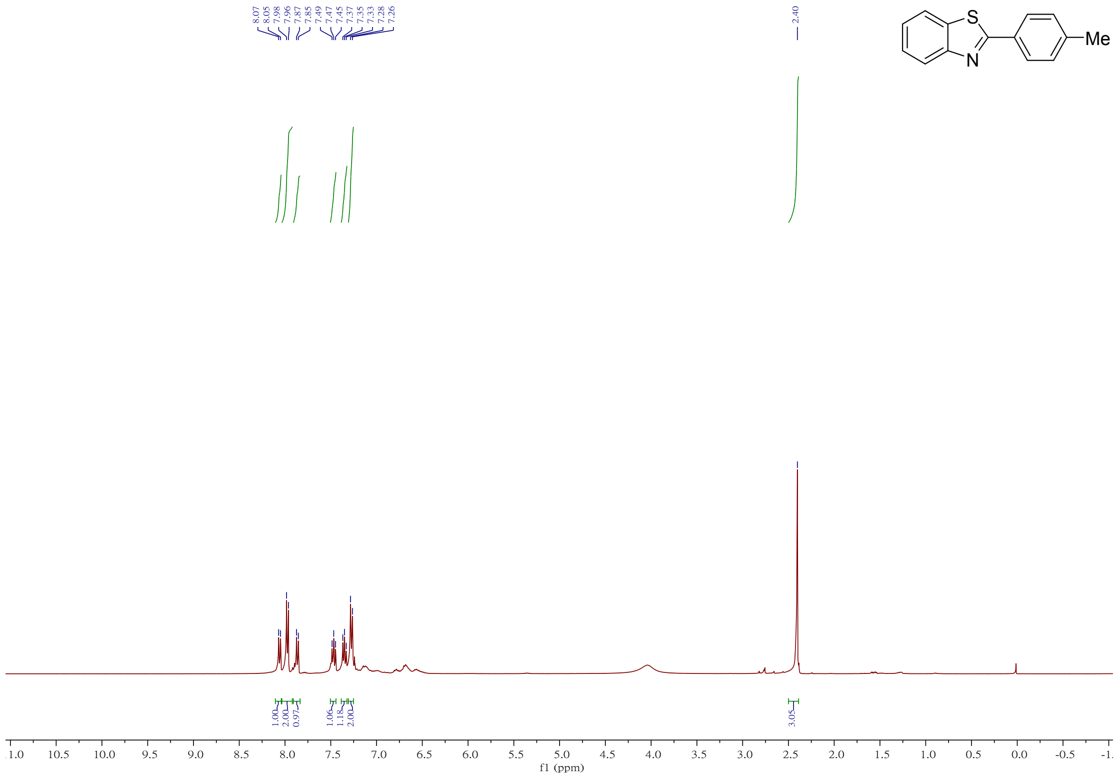


**Figure S12.** Crude $^{1}$H NMR spectrum of compound 3 ($CDCl_3$, 400 MHz).

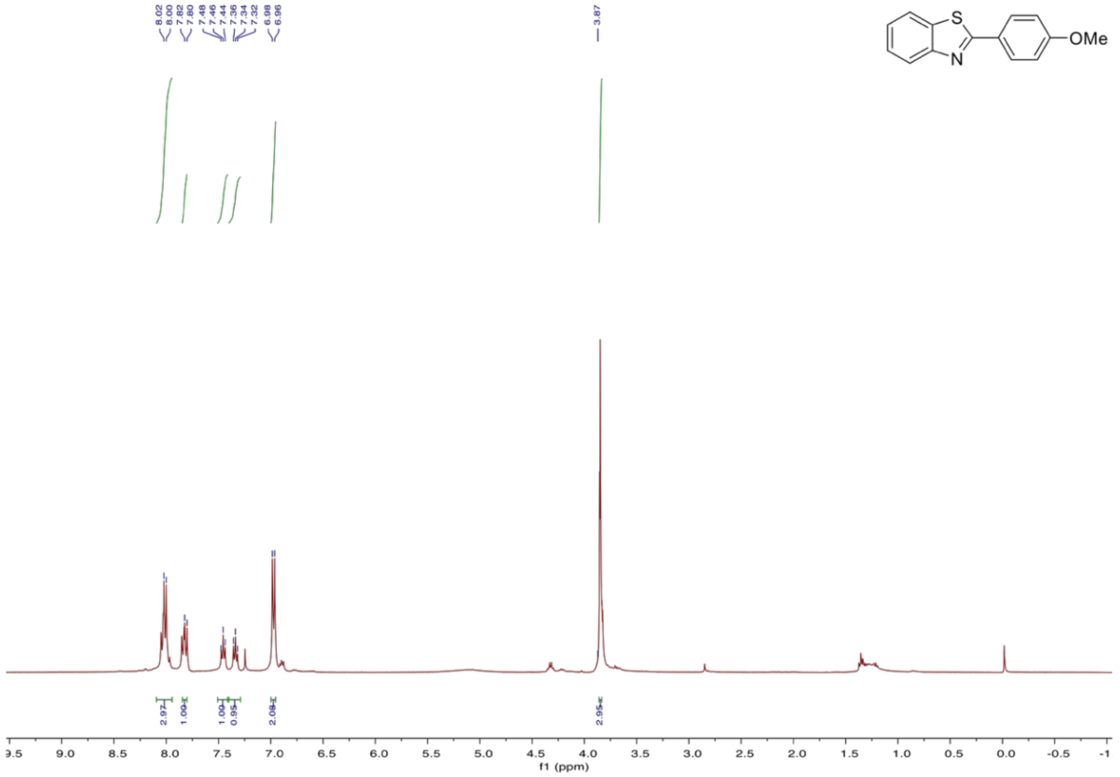


**Figure S13.** Crude $^{1}$H NMR spectrum of compound 4 ($CDCl_3$, 400 MHz).

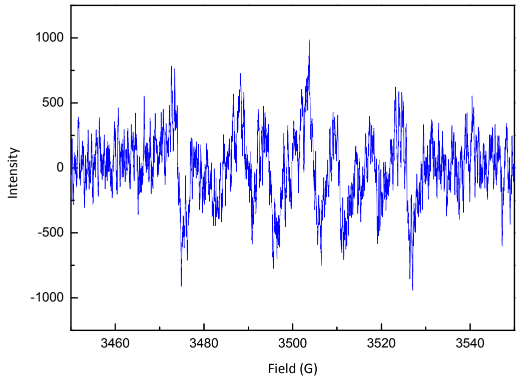


**Figure S14.** EPR spectrum of UV light-irradiated $In_2O_3$ nanocubes in methanol.

**Table S3. Reagent Amounts Used to Synthesize Size-Tunable $In_2O_3$ Nanocubes**

| Reaction vessel | Size | 0.2 M $In(NO_3)_3$ | NaOH | EtOH | T (°C) | T (h) |
|---|---|---|---|---|---|---|
| **Teflon** | **10 nm** | 0.5 mL | 0.03 M 2.5 mL | 7 mL | 180 | 20 |
| **Glass tube** | **72 nm** | 0.2 mL | 0.3 M 3 mL | 6.8 mL | 160 | 2 |
| | **95 nm** | 0.2 mL | 0.3 M 4.5 mL | 5.3 mL | 180 | 2 |

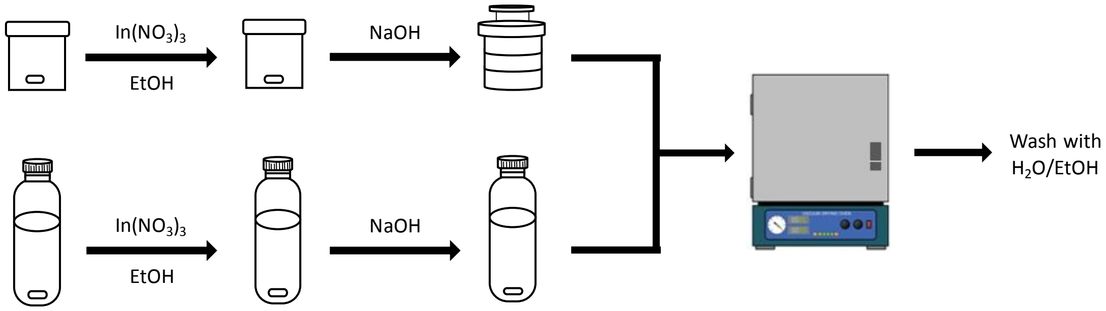


**Figure S15.** Procedure for $In_2O_3$ nanocube synthesis. The Teflon cup has a volume capacity of 25 mL, while that of the glass tube is 30 mL.

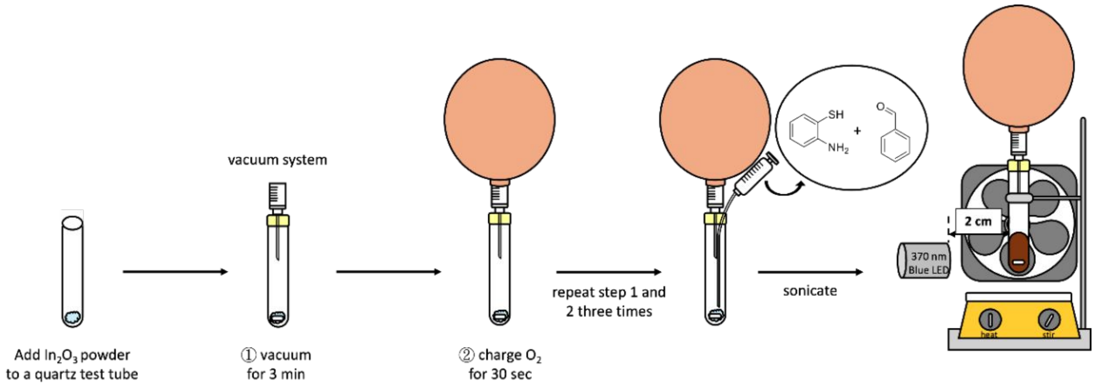


**Figure S16.** Procedure for photocatalyzed synthesis of benzothiazole.
